# Predicted Selective Increase of Cortical Magnification Due to Cortical Folding

**DOI:** 10.1186/2190-8567-2-14

**Published:** 2012-12-17

**Authors:** Markus A Dahlem, Jan Tusch

**Affiliations:** 1 Institut für Physik, Humboldt-Universität zu Berlin, Berlin, Germany; 2 Institut für Theoretische Physik, Technische Universität Berlin, Berlin, Germany; 3 Department of Simulation and Graphics Faculty of Computer Science, University of Magdeburg, Magdeburg, Germany

## Abstract

**Abstract:**

The cortical magnification matrix ***M*** is introduced founded on a notion similar to that of the scalar cortical magnification factor *M*. Unlike *M*, this matrix is suitable to describe anisotropy in cortical magnification, which is of particular interest in the highly gyrified human cerebral cortex. The advantage of our tensor method over other surface-based 3D methods to explore cortical morphometry is that ***M*** expresses cortical quantities in the corresponding sensory space. It allows us to investigate the spatial relation between sensory function and anatomical structure. To this end, we consider the calcarine sulcus (CS) as an anatomical landmark for the primary visual cortex (V1). We found that a stereotypically formed 3D model of V1 compared to a flat model explains an excess of cortical tissue for the representation of visual information coming from the horizon of the visual field. This suggests that the intrinsic geometry of this sulcus is adapted to encephalize a particular function along the horizon. Since visual functions are assumed to be *M*-scaled, cortical folding can serve as an anatomical basis for increased functionality on the horizon similar to a retinal specialization known as visual streak, which is found in animals with lower encephalization. Thus, the gain of surface area by cortical folding links anatomical structure to cortical function in a previously unrecognized way, which may guide sulci development.

## 1 Introduction

**Fig. 1:**
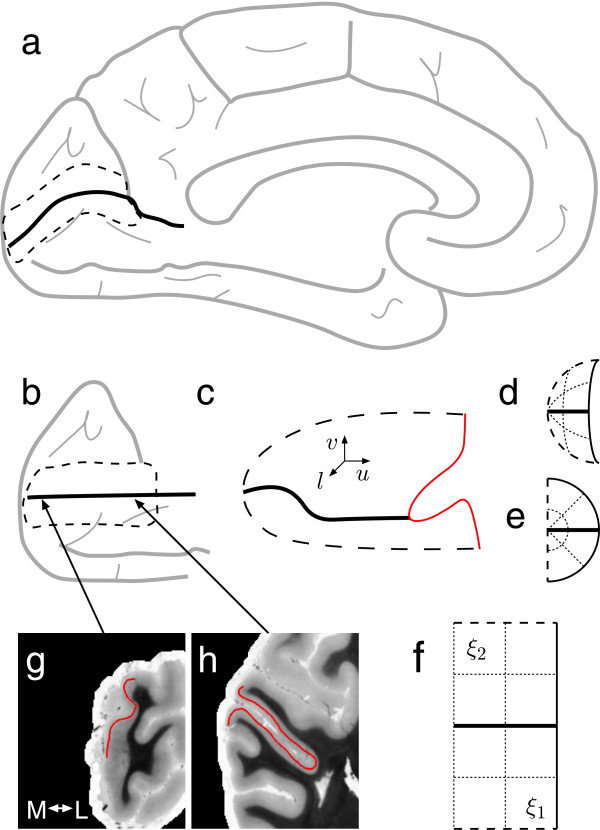
**a** The calcarine sulcus (CS) begins near the occipital pole on the medial surface of a cortical hemisphere and continues toward the posterior end of the corpus callosum (*thick black line*). This is the site of V1. Its border is marked with a *dashed line*. **b** Medial side of the occipital pole, as in **a** but with straight course of CS. **c** The approximate 3D form of V1 in the (u,v,l) coordinates (see text). The representations of the two vertical meridians are *dashed* and the fundus of CS is marked with a *thick black line*. **d** The retinal surface is approximated by a quarter of a sphere, or **e** by a flat disc, as assumed in many experimental data. **f** A Cartesian reference coordinate system of the visual field ($\xi_{1},\xi_{2}$). **g** and **h** Coronal section through V1. The *red line* traces the stria of Gennari. The *M–L axis* indicates the medial to lateral axis

The patterns of the highly folded surface of the cerebral cortex are prominent features of the human brain (Fig. 1a). Primarily, folding permits a larger cerebral cortex surface area to fit inside the skull. However, folding ensures that the additional surface area is not homogeneously distributed, if the surface becomes intrinsically curved. The surface gain is spatially concentrated in certain cortical regions. The cortex represents sensory information in distinguishable fields and therefore the question of the relationship between anatomical structure and sensory function is naturally given. We utilize the methods of continuum mechanics and complex analysis to explore this relationship in the cortex.

Many studies of human cortical architecture show that sensory and motoric fields have some relationship to the gross sulcal and gyral morphology, although a substantial variability in both size and location is observed [1-4]. In a few cases, very precise correlations between sulci and functional entities could be demonstrated. Motor cortex can be identified by the position of the central sulcus [5] and the primary auditory cortex has a clear spatial relationship with Heschl’s gyrus [6,7]. The most reliable relation is, however, the calcarine sulcus (CS) as a landmark of the primary visual cortex (V1) [8-10]. Anatomical identification is also quite reliable for visual areas outside V1, e.g., V5 lies at the intersection of the ascending limb of the inferior temporal sulcus and the lateral occipital sulcus [11,12].

In this paper, we consider V1 not only because of its structural anatomy, but also of its functional retinotopy, i.e., the spatial organization of the neuronal responses to visual stimuli (see below) is well studied in this field [13-18]. The CS, where V1 is located, begins near the occipital pole on the medial surface of a hemisphere. It continues toward the posterior end of the corpus callosum (Fig. 1a). We will present evidence that the 3D form of the CS indicates a selective magnification of the horizon of the visual field that is neither accounted for in standard retinotopic maps nor reflected in the density of retinal ganglion cells, although a modest increase of retinal cell density, the so-called visual streak, can be found [19-21].

Already in 1984, Rovamo and Virsu [22] noted that locally isotropic (independent of the direction of measurement) cortical magnification, that is also symmetric with respect to the meridians, can be better approximated by taking the unfolded convex 3D form of the cortex into account. This does not imply that cortical magnification has to be strictly locally isotropic, but curvature affects the overall layout. Recently, the influence of cortical folding in primate did also take into account the concave folds [23]. We apply tensor analysis to investigate how these symmetries, i.e., isotropy and meridional symmetry, relate to the gross folding pattern, in particular the concave fold of the CS that creates additional cortical space for the representation of the visual field close to the horizon. To this end, we compare intrinsically flat and curved surfaces of V1 and investigate how the 3D form affects cortical magnification. We propose that in particular a horizontal stripe gains additional cortical space. The shape and location of this stripe is similar to the visual streak as a retinal specialization found to be very pronounced in some animals [24-30]. This suggests a link between cortical folding, *M*-scaling and the functional development of cerebral sulci.

First evidence for the predicted selective cortical magnification of the visual horizon can be found in the literature: perceptual filling-in [31] and traveling migraine scotoma [32]. The big advantage of psychophysical methods that measure scotoma is that neither method requires surface reconstruction to measure cortical magnification. But it is difficult to get reliable quantitative data, because these methods involve psychophysical investigations with subjective evaluations from probands and patients. Furthermore, there is a brief report of such a phenomenon [33]. Only computationally-intensive methods, which allow precise surface-based morphometry using anatomical magnetic resonance imaging and functional magnetic resonance imaging (MRI and fMRI) [18,34,35], can provide a direct test of our predicted correlation between CS, as an anatomical cortical landmark, and increased *M*, as a functional cortical measure.

## 2 Material and Methods

We use concepts from continuum mechanics to describe the retino-cortical map [36]. In the Lagrangian description of continuum mechanics, the map is seen as a deformation rather than a transformation. This description uses some coordinate system in the sensory space as a reference configuration to express every quantity in the deformed configuration, i.e., as the cortical map of the sensory space. The deformation language is intuitive but needs to be adapted for our purposes. We emphasize that we take mostly a kinematic approach. No attention is paid to the dynamics of the creation of a retinotopic map other than that we compare two discrete stages of V1, one which is intrinsically flat and one that evolved from the latter such that it is intrinsically curved with a major sulcus. The Lagrangian description is also suited for a dynamical description continuous in time of map development, for example with self-organizing neural networks [37].

Let us start by briefly clarifying the terminology, in particular related to cortical mapping and curvature; linear and areal cortical magnification; the naming of the visual coordinate system; and, finally, tensor versus matrix methods.

Conformal maps allow us to describe cortical magnification by a scalar field, simply referred to as cortical magnification factor. In fact, we start to consider conformal maps between domains in the complex plane, that is, we consider intrinsically flat domains. Conformal maps also exist between intrinsically curved domains. Note that throughout the manuscript, we do not consider extrinsic curvature unless we say so. Of course, to fit larger cerebral cortex surface area inside the skull, nature can make primarily use of extrinsic curvature, but not exclusively (unless the cortex would be a cylindrically scrolled structure). In the following, we will refer to V1 as being curved (“curved V1”) if it is a surface with intrinsic curvature at least at some locations within V1, i.e., locations that have a nonvanishing Gaussian curvature. As a consequence, a curved V1 must be embedded in 3D even if it is mostly flat. And we will refer to V1 as a “flat V1” if it is an intrinsically flat surface, i.e., the Gaussian curvature is zero everywhere.

It can be important to distinguish linear and areal cortical magnification if we assume *M*-scaling, i.e., the fact that a measured quantity remains qualitatively similar across the entire visual domain when magnified in inverse proportion to *M*. For example, the time required for a scotoma of certain size to fade and become replaced by its background (perceptual filling-in, see discussion) can be proportional either to linear or areal cortical magnification. The underlying mechanism could involve merely the scotoma diameter, which would suggest linear *M*-scaling or involve the total area covered by the scotoma, which would suggest areal *M*-scaling. The term *M*-scaling is unfortunately sometimes used without mentioning which is meant. Note that the distinction between linear or areal cortical magnification is in the cases where *M* is a scalar field not essential as these factors can easily be converted, but one still must specify which method was used and in which units *M* is given (see next section).

To specify loci in the visual hemifield, a polar coordinate system with its origin in the viewer’s fixation point is used (Fig. 1e). The coordinate lines at fixed polar angles are called meridians, with the horizontal meridian (HM) as the reference set to be zero degrees (bold solid line in Fig. 1e). Meridians increase and decrease in the upper and lower visual field quadrant to 90 degrees going anticlockwise and clockwise, respectively, until the two vertical meridians (bold dashed lines in Fig. 1e) bound the visual hemifield.

We will express the generalized cortical magnification in terms of a matrix ***M***. We want to stress that cortical magnification is a concept independent of the retinal and cortical coordinate systems chosen to represent it. Therefore, ***M*** actually is a cortical magnification tensor. It may lead to some confusion, when we later introduce ***M*** based on the cortical metric in the sensory coordinate system. We need to consider some sensory coordinate system, which merely reflects the fact that cortical magnification is not exclusively a property of the intrinsic geometry of the cortex, but of the sensory map in the cortex. In mathematical terms, ***M*** is the square root of the matrix expressing the Riemannian metric of V1 in the sensory polar coordinates.

The Lagrangian formulation of such maps is a tensor approach. As an alternative to Riemannian geometry, it provides by definition some retinal parameterization of the cortical surface that is needed for the concept of magnification from retina to cortex. In contrast, the cortical metric in an arbitrary parameterization can be used, for example, to define the Laplace–Beltrami operator as a generalization of the Laplace operator and much effort has gone in the development of computationally advantageous parameterizations in this regard. However, since the cortical magnification is usually discussed referring to meridians and eccentricity, it is natural to use exclusively this polar coordinate system. Therefore, we refer to the generalized ***M*** as the cortical magnification matrix and avoid the potentially daunting term tensor.

## 3 Results

We will first define the contour of an intrinsically flat V1. Then we will define the cortical magnification matrix and use this concept firstly to compare cortical magnification along different radial directions (from horizontal to vertical) on the flat V1 and show that the vertical direction has an increased cortical magnification. Let us emphasize that retinotopic maps that are assumed to be intrinsically flat can still be extrinsically curved. We will define an intrinsically curved V1 starting from an extrinsically flat V1 by plausible deformations. In the second step, the retinal coordinate system is defined on this curved V1, preserving some symmetry constraints while relaxing others. Finally, we use the concept of cortical magnification matrix to predict a selective increase of cortical magnification along the horizon due to cortical folding.

### 3.1 Symmetries in the Retinotopic Map

**Fig. 2:**
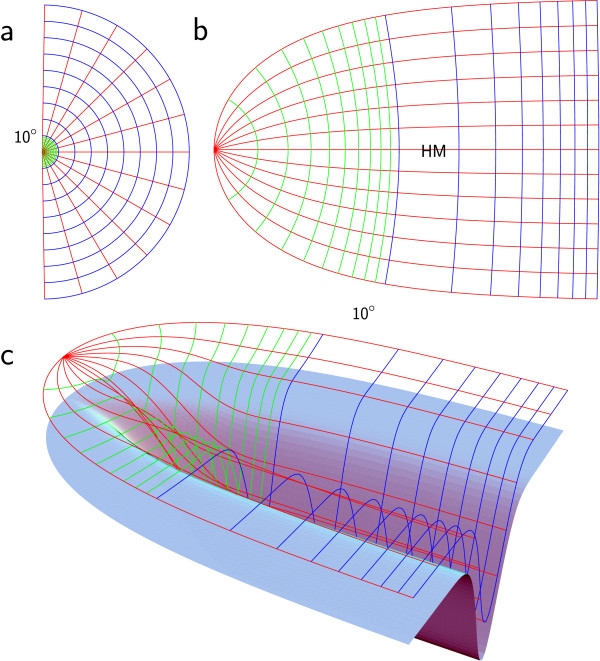
**a** Retinal grid which serves as a reference frame in the Lagrangian formulation of retinotopy. In the inner 9^∘^, iso-eccentricity lines are drawn in 1^∘^-steps in *green*. From 10^∘^ to 90^∘^ iso-eccentricity lines are drawn in 10^∘^-steps in *blue*. Meridians are drawn in 15^∘^-steps in *red*. **b** The neural representation of the retinal grid mapped with Eq. 3 serves as a flat model of V1. **c** The *blue surface* serves as a curved model of V1. It is created by forming **b** with lift functions (see Fig. 1g and h) in the third dimension. The neural representation of the retinal grid on this surface is placed such that meridians are equidistantly spaced

As a starting point, we need the contour of the area of V1. The contour of V1 can be defined by the bounded domain of the visual hemifield that V1 neurally represents. Simply speaking, V1 has a map of one visual hemifield with a nonuniform map scale. This scale is called cortical magnification. This map, or rather the retinotopic mapping function, governs the contour of V1 because it maps the bounded domain of the visual hemifield, approximated by a half disc, that extents to a visual eccentricity of nearly up to 90^∘^ (see Fig. 2a). We will contrast some empirical observations with deductive reasoning based on plausible symmetry conditions on the retinotopic map to obtain the contour of V1 and to introduce these symmetries that guide us through the following sections.

The retinotopic map is usually expressed by the cortical magnification factor *M*. For a mapping function from a one-dimensional domain (a line) to another, e.g., along a single meridian, it is sufficient to know the value of *M* on this domain. Note, that the term *linear* cortical magnification factor (see previous section) refers to the one-dimensional domain. This can cause confusion because *M* is often modeled as an inverse linear function of a single coordinate, the retinal eccentricity *θ*, the fact that will be important in the following brief analysis of the involved symmetries.

In the one-dimensional case, *M* is simply a derivative of the mapping function. The mapping function can therefore be determined up to a constant of integration. This constant can be set to zero because it describes only a translational shift.

The complex logarithm provides a standard formulation of retinotopy in a flat V1. Fischer [38] first suggested this analytic function for the transformation of a visual field into its neuronal representation. While this relation was derived from visual inspection of ganglion cell density and receptive field size distribution, assuming these quantities are *M*-scaled, it is supported by data of M(θ)[39], as shown by Schwartz [40] with a power law $\frac{1}{b}\theta^{-n}$ fit, the exponent *n* being sufficiently close to unity to be replaced by it. The meaning of parameter *b* will be explained below. Thus, the integral equation reads as

(1) $$u(\theta )=\int M(\theta )\;d\theta =\int \frac{1}{b}\theta^{-1}\;d\theta =\frac{1}{b}\log\theta ,$$

where *u* is a cortical linear distance, for instance, the distance along the cortical representation of the horizon in the visual field.

In two dimensions, the situation is more complicated. The exact unity value of the exponent n=1 can even be postulated ab initio from symmetry constraints for 2D maps. It is therefore important to distinguish these two perspectives. In 2D, a possible but purely hypothetical function is obtained by generalizing the real function in Eq. 1 to a corresponding complex function

(2) $$w=\frac{1}{b}\log z.$$

The magnitude of *z* is the retinal eccentricity *θ* and its argument *ϕ* is the azimuth ($z=\theta e^{i\phi }$), and the real and complex parts of *w* are Cartesian coordinates (u,v), respectively. Generalizing the real function in Eq. 1 to a corresponding complex function implies two rather obvious constraints on the retinotopic map and one more subtle constraint.

Firstly, a complex function implies a conformal mapping, that is, cortical magnification becomes a scalar field. This is a symmetry, namely that cortical magnification at any location is invariant with respect to direction. Therefore, cortical magnification is locally isotropic. At least for some *M*-scaled visual functions, this seems to be a natural symmetry requirement, such as for visual acuity. Secondly, complex functions are conformal maps between domains in the complex plane, that is, intrinsically flat domains, which, of course, can still be extrinsically curved to accommodate the limited extend of the skull. This is another symmetry, namely that the Gaussian curvature is constant and zero everywhere.

These symmetries, which are given by the constraints of analytical functions, look rather reasonable. But even if these symmetries were reasonable in the light of missing or uncertain experimental data (see discussion), they should not be given preference to further symmetries that could also be found in the retinotopic map, in particular meridional symmetry of cortical magnification, i.e., *M* is invariant under retinal rotations around the center.

We will discuss whether the meridional symmetry of *M* should be considered as a plausible constraint, but let us end this section by emphasizing its severe consequences.

The real and imaginary parts of an analytic function are conjugate harmonic functions that solve Laplace’s equation. From Laplace’s equation in polar coordinates, it can be easily shown that Eq. 2 is harmonic and that any harmonic function that depends only on the eccentricity *θ* must be of this form. Alternatively, this follows from Cauchy–Riemann differential equations in complex analysis, which must be satisfied if we assume that the analytical mapping function is differentiable [41].

Thus, it is readily shown that any meridional symmetric analytic function must be of the form of Eq. 2. Therefore, any retinotopic map that is an analytic function with a meridional symmetric magnification factor *M* implies that cortical magnification is an inverse linear function (in fact, a linear function with the axis intercept at zero). So, if cortical magnification is investigated under the following three assumptions: (a) independent on direction (conformal map), (b) intrinsically flat (with (a) this leads to analytical functions), (c) meridional symmetry of *M*, one should be aware of the implicit assumption of inverse linear cortical magnification in the form of Eq. 2.

Even more importantly, Eq. 2 fails to describe the retino-cortical map close to the representation of the fovea (θ<1) because the foveal point at θ=0 is a singularity. To include the foveal region, an offset can be introduced

(3) $$w=\frac{1}{b}\log\left(\frac{b}{a}z+1\right)$$

together with another parameter *a*. Equation 3 is not meridionally symmetric. It follows from the reasoning before that a retinotopic map that is based on an analytic function and includes the representation of the fixation point (θ=0) cannot be meridionally symmetric.

Both parameters can now be interpreted easily when Eq. 3 is differentiated: $\frac{1}{a}$ is the value of the linear cortical magnification factor *M* at the center of the visual field in terms of millimeters of cortex per degree of visual angle. The parameter *b* is the linear growth rate (slope) of the inverse linear cortical magnification factor on the real axis (ϕ=0), i.e., along the horizon. This map is called a monopole map (see Fig. 2a–b). Values of the parameters *a* and *b* are given in the literature [42]. Note that sometimes different parameters are used, in particular $w=A\log(z+E_{2})$ with $a=E_{2}/A$ and b=1/A.

We use normalized values in units of *a*. The value of *b* is chosen as 0.57265*a*, which would correspond to a=0.117 and b=0.067 as in [43]. When Eq. 2 is used, for example, by [18], there is only parameter *b*. Our dimensionless parameter *a* can be estimated from the contour of the flat map.

The inverse linear magnification holds in the map given by Eq. 3 only at the horizon. Due to the shift, none of the other meridians take a simple course in the complex domain (overlapping with coordinate lines of the real and imaginary part or the coordinate lines of the absolute value and argument). Therefore, to investigate the cortical magnification factor along other meridians, we introduce the cortical magnification matrix, which can express magnification at arbitrary points along arbitrary directions most easily. Furthermore, unlike the scalar factor *M*, this matrix is also suitable to describe anisotropy in cortical magnification, which is of particular interest in the highly gyrified human cerebral cortex. But it can also be useful for conformal maps, in which case the matrix at any point can be transformed to the identity matrix multiplied by a scalar.

### 3.2 Generalized Cortical Magnification

The natural description for a generalized cortical magnification is the Lagrangian description. It uses the retinal configuration as a reference to express every quantity in the cortical configuration. Mathematically this is expressed as

(4) $$\Phi :D_{R}\to D_{V1}:r\mapsto p=\Phi (r),$$

where *r* and *p* are points in the retinal and cortical domain $D_{R}$ and $D_{V1}$, respectively. The Jacobian matrix of the map *Φ* can be interpreted as a deformation gradient

(5) $$J_{\Phi }(r)=\nabla \Phi (r).$$

It is a homogeneous transformation tangent to the transformation *Φ* attached at the image of *r*. Caution is needed when the classical linear cortical magnification factors are derived from the components of $J_{\Phi }$. Usually non-Cartesian coordinates are utilized to describe the retinal reference state. Furthermore, $J_{\Phi }$ contains a rotation which carries the retinal directions onto its neural representations. This rotation introduces problems if cortical magnification is not isotropic. To obtain the linear cortical magnification factor in accordance with its definition [39], one must use the scalar product of a vector in the considered direction. The linear cortical magnification factor $M_{v_{r}}$ of a retinal tangent unity vector $v_{r}$ ($∥v_{r}∥=1$) attached at *r* is the norm of its cortical image under the homogeneous transformation:

(6) $$M_{v_{r}}=∥\nabla \Phi (v_{r})∥.$$

This norm and the underlying scalar product is defined via the cortical metric in retinal parameterization

(7) $$g_{V1}=J_{\Phi }^{T}g_{R}J_{\Phi },$$

where $g_{R}$ and $g_{V1}$ are the metrices of retina and cortex, respectively. The cortical metric $g_{V1}$ leads to the definition of the cortical magnification matrix. This matrix is founded on a notion similar to that of the linear cortical magnification factor *M*, but is much broader in conception. Once we have this matrix, we can calculate the linear and cortical areal magnification factors on the flat and curved model of V1 and compare the results.

In continuum mechanics, $g_{V1}$ is called the right Cauchy–Green tensor and termed ***C***. For the sake of simplicity, we follow p. 44 in Salencon [36] and adopt the term (cortical) expansion tensor for ***C***. This name may better convey the meaning of this matrix. Its eigenvalues and eigenvectors give the shape and orientation of an ellipsoid representing an initially spherical infinitesimal area in the retina. Furthermore, this alternative name reminds us that we are not interested in the cortical metric expressed in an arbitrary coordinate system, but in one that is related to the sensory space. This would be sufficient for other surface-based 3D geometric tasks using, for instance, the Laplace–Beltrami operator to create flat maps with minimal surface area distortions. Using this particular cortical surface parameterization, namely some sensory coordinates, makes the choice fundamental. It links the metric to sensory function and it allows us to describe changes of the cortical surface from the sensory perspective.

The square root of ***C*** is a pure stretch tensor that is directly related to the classical concept of cortical magnification and it could therefore also be termed cortical magnification tensor, yet the term matrix is probably more intuitive in the neuroscience community

(8) $$M=\sqrt{C}.$$

The matrix ***M*** is alternatively obtained from a polar decomposition of $J_{\Phi }J_{\Psi }$. The Jacobian matrix $J_{\Psi }$ is the homogeneous transformation tangent to *Ψ*, which maps Cartesian coordinates to the visual field coordinates, i.e., either spherical or polar (retinal) coordinates. The distinction between spherical and polar coordinates should be formally made, since data of the visual field position can be obtained either with perimetry or campimetry. We can avoid this, however, if we transform the retinal system into the Cartesian coordinates $\left(\xi_{1},\xi_{2}\right)$ of the visual field (see Fig. 1g), because data is often available in these coordinates, for instance, migraine aura scotoma drawn on paper and subsequently scanned.

### 3.3 Linear Cortical Magnification Factor Derived from the Matrix ***M***

The linear cortical magnification factor $M_{v_{r}}$ can be defined at any point *r* for arbitrary directions ***v***. In experiments, $M_{v_{r}}$ is usually measured along a constant meridian, though not necessarily along this direction. In conformal maps, e.g., the monopole map (Eq. 3), $M_{v_{r}}$ depends only on position *r* but not on direction ***v***.

**Fig. 3:**
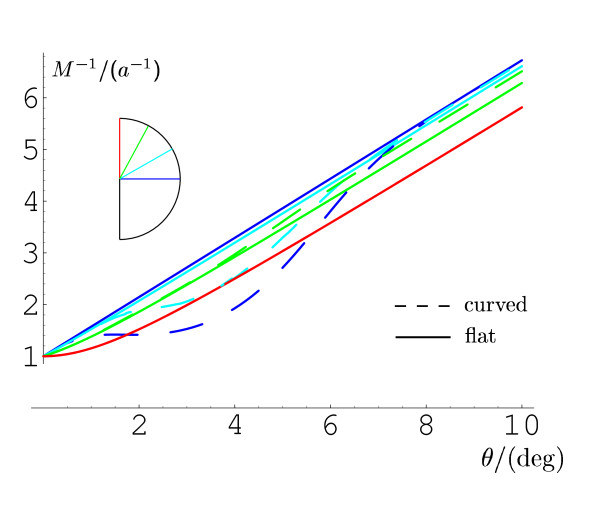
Inverse linear magnification factor as a function of eccentricity *θ* along different meridians (horizontal meridian (HM) 0^∘^: *blue*; 30^∘^: *cyan*; 60^∘^: *green*; vertical meridian (VM) 90^∘^: *red*, see *inset*). *Solid lines* are on the flat model, *dashed* on the curved model of V1. *M* has an inverse linear dependence on retinal eccentricity *θ* only along the neural representation of HM on the flat model of V1 (*solid blue line*). While on the flat model of V1, *M* increases with azimuthal distance at constant eccentricity (*solid lines*). This meridional asymmetry is reversed on the curved model of V1 (*dashed lines*). In particular meridians close to HM gain cortical surface on the 3D model of V1

We compare the $M_{v_{r}}$ on the monopole map (flat V1) with the one on the curved V1 along the *θ*-direction. This direction is most frequently used in the definition of the classical linear cortical magnification factor. Therefore, we will use *M* without any index for linear magnification in this direction. It is convenient to plot the inverse linear cortical magnification factor because this is a linear function in *θ* on HM in the monopole map. As the meridians in the monopole map change from horizontal to vertical, the inverse linear cortical magnification factor grows slower than linear (see Fig. 3). This is readily understood, considering the layout of the visual hemidisc mapped by Eq. 3 into its neuronal representation (Fig. 2b). While the representations of all meridians extend approximately equally long into the cortical *u*-direction (in anatomical terms the posterior–anterior axis), the more vertical they are, the larger is their evasion into the cortical *v*-direction (into the dorsal and ventral direction for the upper and lower visual quadrant, respectively). The increase in the cortical space on the vertical meridian (VM) as compared to HM is about 20 %. While in fact one study indicates the opposite asymmetry with HM being cortically over represented with respect to the vertical meridian [33].

### 3.4 3D Model of V1 with Curved Retinotopy

We construct the 3D model of V1 based on our own and published data obtained at autopsy of neurologically normal human brain [8,10]. The dimensions of V1 were determined from cross-sections in both hemispheres. The surface of V1 can easily be identified postmortem by the stria of Gennari. This band of myelinated axons can be traced (see Fig. 1g–h). Such traced curves provide lift functions l(u,v) for forming the originally flat monopole map (Eq. 3) into its 3D from. We will only summarize the data obtained by visual inspection of the stria of Gennari used to define plausible functions that define the 3D form. In particular, we outline the restrictions we impose regarding plausible symmetry principles.

First, we transform the complex function in Eq. 3 into the real $R^{2}$ domain

(9) $$\begin{array}{rl} u(\theta ,\phi ) & =\frac{1}{2b}\log\left(\frac{a^{2}+2b\theta \cos(\phi )a+b^{2}\theta^{2}}{a^{2}}\right),\\ v(\theta ,\phi ) & =\frac{1}{b}\arctan\left(\frac{b\theta \sin(\phi )}{a+b\theta \cos(\phi )}\right). \end{array}$$

Equation 9 describes a (θ,ϕ)-parameterized surface in $R^{3}$ together with the lift function l(u,v), that is, in the Cartesian coordinates (u,v,l). Note that the lift functions are used in the first step to describe the surface, but not the retinotopy on it. We define lift functions in such a manner that the initially flat surface becomes intrinsically curved. Note that we may introduce a bias since we actually treat the intrinsically flat surface also as extrinsically flat at this step of the construction. But the bias does not necessarily affect retinotopy, because we rearrange the location of the meridians. Applying the lift function directly to the retinotopic grid (Fig. 2b) would drive some adjacent meridians farther away from each other than others, depending on $\frac{\partial l(v,u)}{\partial v}$. Therefore, to obtain a curved retinotopy, we need to rearrange the location of the meridians by an inverse sampling technique, as will be described at the end of this section.

V1 is located entirely or nearly entirely on the medial surface of the occipital lobe. We align parallel to this surface the (u,v)-plane and define the lift functions l(u,v) such that about two-thirds will lie within the CS walls. Furthermore, l(u,v) depends on the steepness of the walls of CS and on its course in the (u,v)-plane (see Fig. 1a). Due to the large variations among individuals, we have to make some simplifications. We assume that the course of CS can be deformed to follow a straight course.

To finally construct the lift functions l(u,v), it is useful to define another landmark: the fundus of CS (FCS). Roughly speaking, it is the curve of maximum depth that spans the length of the CS. A more precise definition can be given based on curvature or based on distance functions [44]. Based on FCS, the assumption that the course of CS is straight can be formulated in a different way. In this case, the FCS assumes a curved line without torsion and its projection onto the (u,v)-plane is a straight line (see Fig. 1c). For simplicity, we assume that CS has a bilateral symmetry with the symmetry plane going through this line. Furthermore, the mathematical description of V1 is w.l.o.g. simplified if the projection of FCS onto the (u,v)-plane lies on the *u*-axis. Then the family of parametric profiles $l_{u}(v)\equiv l$ (u=const., *v*) in the coronal planes ${\left(v,l\right)}_{u}$ completely describe the 3D-configuration of V1.

It significantly simplifies the later performed resampling to define the lift functions along curved coordinate lines in the (u,v)-plane with constant *θ*, instead of along the straight coordinate lines with constant *v*. Since the family of parametric profiles ${\tilde{l}}_{\theta }(\phi )$ live in curved planes, we transform them into a Cartesian $\left(\xi_{1},\xi_{2}\right)$ plane (see Fig. 1f). This results in a family of parametric profiles ${\tilde{\tilde{l}}}_{\xi_{1}}(\xi_{2})$, which we finally choose to be Gaussian-shaped

(10) $${\tilde{\tilde{l}}}_{\xi_{1}}(\xi_{2})=de^{-\xi_{2}^{2}/\sigma_{\xi_{2}}^{2}}.$$

The parameter $\sigma_{\xi_{2}}$ defines the width of CS. Its value is $\frac{\pi }{10}$ (see Fig. 2c). The parameter *d* gives the depth of FCS as a function of the new eccentricity coordinate $\xi_{1}$ in the $\left(\xi_{2},l\right)$-plane. Again, we choose a Gaussian-shaped profile

(11) $$d(\xi_{1})=\frac{d_{\max}}{s}\left(1-e^{-\xi_{2}^{2}/\sigma_{\xi_{1}}^{2}}\right).$$

The parameter $\sigma_{\xi_{1}}$ defines the steepness of CS. Its value is $\frac{\pi }{50}$ (see Fig. 2c). The parameter $d_{\max}=170.94$ gives the maximal depth of CS in units of *a*, equivalent to 20 mm. The factor $s=\frac{1}{\pi }\int_{-\pi /2}^{\pi /2}\sqrt{1+{\left(\frac{du}{d\theta }\right)}^{2}}\;d\theta$ is needed to resize the profiles, since we define the lift function on the $\left(\xi_{1},\xi_{2}\right)$ plane but want to have a Gaussian-shaped FCS in the (u,l) plane. Each arc with constant eccentricity spans an angle *π* in the visual field. On the $\left(\xi_{1},\xi_{2}\right)$ plane these arcs have all the same length, while on the (u,v)-plane their arc length is *πs*. Therefore, we have to resize $l_{\xi_{1}}(\xi_{2})$ by this quotient.

**Fig. 4:**
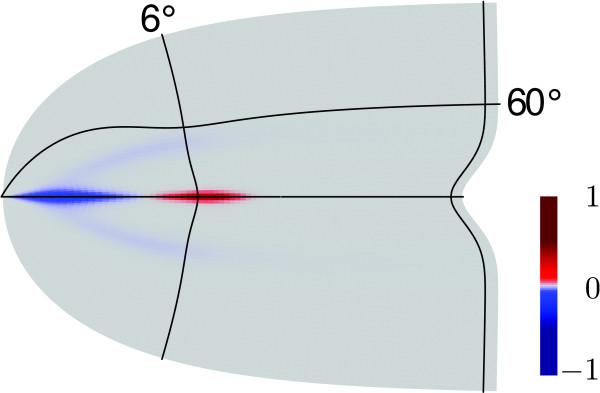
The Gaussian curvature on the curved model of V1 (cf. Fig. 2c)

The Gaussian curvature *K* is determined by this construction because it depends only on the metric (the retinotopy depends on the metric in a retinal coordinate systems). In Fig. 4 we show *K* in a color code on this surface. It is nearly everywhere close to zero, except for two main locations. One is at the saddle-shaped entrance of the FC. Here, *K* is negative (blue). Father along the fundus, when CS reaches its maximal depth, *K* changes to positive values (red).

To summarize, four hypotheses have been make about the area V1: (i) two-thirds will lie within the CS walls, (ii) the walls are symmetric with respect to the fundus, (iii) the shape of the walls can be approximated with smooth Gaussian-shaped profiles with constant standard deviation, and (iv) the shape of the fundus can also be approximated with smooth half Gaussian-shaped profile (we have alternatively chosen cosine-shaped profiles for both walls and fundus, with almost identical results).

### 3.5 Retinotopy on a 3D Model of V1

To compare retinotopy on the flat and curved model of V1, we now define the retinotopic grid on the latter. Although this may seem obvious, we want to note that we actually define the retinotopy on the curved 3D surface by this.

Firstly, assume that HM maps to the fundus of the curved model surface. By definition HM splits the retinotopic map into two quarter fields, a dorsal (v>0), lower field (ϕ<0), and a ventral (v<0), upper field (ϕ>0). If we simply lift the neural representations of the meridians from the flat surface model of V1 onto the dorsal and ventral part of the curved model by Eq. 10, the meridians drive away from each other in proportion to $\frac{\partial l(v,u)}{\partial v}$. In fact, meridians should by equidistantly spaced in the curved model of V1, as they are on the flat surface (see red coordinate lines in Fig. 2c). This can be achieved by rearranging *ϕ* on the flat model of V1 before lifting with the inverse sampling equation

(12) $$\phi (\tilde{\phi })=\ell_{\theta }^{-1}\left(\frac{2\tilde{\phi }-\pi }{\pi }\ell_{\theta }\left(\frac{\pi }{2}\right)\right).$$

The function $\ell_{\theta }(\phi )$ gives the arc length of the profile $l_{\theta }(\phi )$ between the origin (ϕ=0) and the value of the argument *ϕ*. $\ell_{\theta }^{-1}$ is the inverse function with the new value of *ϕ* as the return value. The rearranged meridians are then lifted down from the flat model with a lift function (Eq. 10). Finally, we need to place the iso-eccentricity coordinates, i.e., the green (inner 10^∘^) and blue (10^∘^–90^∘^) coordinate lines in Fig. 2c. They are lifted from the flat model with the lift function (Eq. 10) without rearrangement.

### 3.6 Areal Magnification and Surface Area Gain

**Fig. 5:**
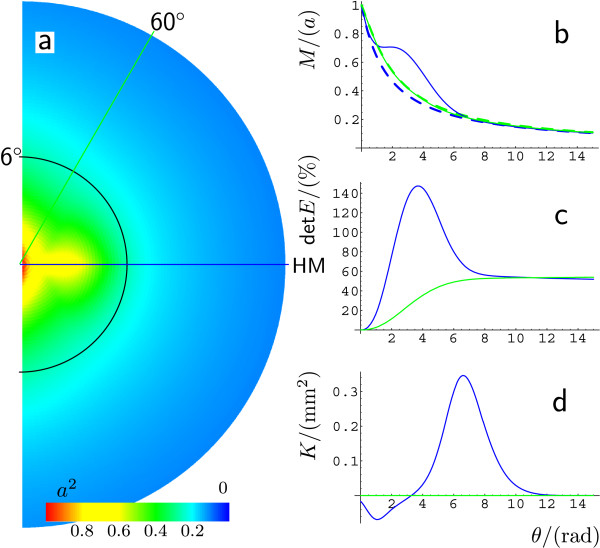
**a** Areal cortical magnification factor det***M*** projected onto the visual hemifield. The *color bar* indicates the normalized value in units of $a^{2}$. The *yellow blob* at an eccentricity $\theta =3^{\circ }$ along the horizon indicates the virtual visual streak. **b** Linear cortical magnification *M* vs. eccentricity *θ* in units of *a* along the horizontal meridian at $\phi =0^{\circ }$ (HM) and the meridian at $\phi =60^{\circ }$ (*dashed* and *solid lines* on flat and curved model of V1, respectively). **c** Normalized surface area gain det***E*** and **d** Gaussian curvature along HM and $\phi =60^{\circ }$

Figure 5a shows the areal cortical magnification factor projected in the visual hemifield for the curved V1. This factor predicts the retinal ganglion cell density, assuming this quantity is *M*-scaled [20]. There is a larger value of det***M*** on a stripe centered around HM. The inverse profile of M along this stripe is visualized in Fig. 3. The graph of *M* in Fig. 5b visualizes this stripe as a shoulder in the exponentially decreasing curve M(θ) at HM. The shape and location of this stripe in the visual field (see Fig. 5a) are similar to a retinal specialization known as a visual streak. The visual streak is a stripe of elevated neuronal density along HM in the retina. It is found in some animals [24-30] but it is not pronounced in humans [19-21]. The stripe centered around HM can be thought of as a virtual visual streak as described in the following.

The neuronal density in the retina is assumed to be M-scaled (areal magnification). Let us define a virtual counterpart of the visual streak as the elevation of inversely retinotopic projected densities of cortical cells. Along the horizontal meridian, the retinal representation of cortical cells is largely elevated with respect to other more vertical meridians outside this visual streak. A virtual visual streak is related to surface area magnification, but also to surface gain by folding. Just as Eq. 6 defined the stretch of a unity vector, we can calculate the surface area gain of a unity area in the retina via the expansion tensor ***C***. Considering rigid body transformations, ***C*** is the unity tensor **1** and surface area gain is a measure that must be zero. This led us to define the strain tensor $\varepsilon =\frac{1}{2}(C-1)$. In continuum mechanics ***ε*** is called the Green–Lagrange strain tensor. The tensor ***ε*** is a measure of surface gain from retina to cortex. The measure of surface area gain by cortical folding is obtained when the retinal reference frame is replaced by its neural representation on the flat model surface of V1. The strain tensor of the curved model of V1, with the cortical flat reference frame, is called ***E***. It measures the surface gain by folding.

The value of det***E*** corresponds to the elevation of retinal density that is needed to satisfy *M*-scaling in a folded cortex. To show that this is in good agreement with some experimental data [31,32] (see also discussion), we look at det***E*** along different meridians (Fig. 5c). For all meridians, folding provides equally increased cortical space for the representation of the periphery of the visual field. This increase is mainly limited to the periphery because the calcarine sulcus just starts at the neural representation of the fovea and additional surface area is mainly gained when the calcarine sulcus has reached its maximum depth. Close to the fovea, meridians close to HM also gain cortical surface area for their neural representation (see peak in Fig. 5c at HM).

The location of the virtual visual streak, i.e., increased values of det***E***, depends on the particular form of V1. Our 3D surface model of V1 averages over the observed substantial variability [8-10]. A statement about the location of the virtual visual streak independent of this variability can be done when we describe the location of the virtual visual streak relative to the curvature of V1. For this purpose, one needs to consider the Gaussian curvature, because it is determined only by intrinsic properties of the surface. The Gaussian curvature of a point on a surface is the product of the maximal and minimal curvature of all curves passing through this point. As a consequence, a saddle-shaped surface has negative Gaussian curvature while a spherical surface is positively curved. The entrance of CS is saddle-shaped and has therefore negative Gaussian curvature. The location where the fundus of the calcarine sulcus reaches its maximal depth has positive Gaussian curvature. The peak of the virtual visual streak is located at the transition between negative and positive Gaussian-curved areas (Fig. 5d). While this provides only a rough estimate of the location of a virtual visual streak, it is not only independent from the variability, but can also be used as a prediction for selective magnification in other areas in the cerebral cortex.

## 4 Discussion

Cortical magnification is the local description of retinotopy or, in fact, of receptotopic maps in general. Tonotopic and somatotopic maps are other prominent examples [5-7] for which cortical magnification can be defined in the same manner as for the visual modality. Cortical magnification is the most fundamental quantity of cortical feature maps that describes how much cortex is devoted to processing sensory information. A thorough mathematical foundation of magnification is needed if we want to describe anisotropies and other symmetry breaking constraints in such maps that may serve distinct purposes in sensory information processing. We will start our discussion with experimental evidence supporting our predictions, continue with methods able to verify them, and provide the context to current alternative tensor methods. Then the limitation in symmetry properties of flat models of retinotopy and our assumptions concerning the 3D surface model of V1 are discussed. We finish with conclusions that can be drawn from our results on sulcal development.

### 4.1 Experimental Studies Supporting Meridional Asymmetry

The approximate form of human retinotopy has long been established from various methodologies. Some methods obtain *M* indirectly, they measured a quantity that is assumed to be *M*-scaled. However, these methods were only advantages before non-invasive imaging became available. If *M* is known at various loci and also additional constraints are given, such as certain symmetries, one can determine the layout of the global retinotopic map. Psychophysical studies that obtain an *M*-scaled quantity provide an elegant measure of cortical magnification mainly because they do not require an explicit reconstruction of the cortical surface. They greatly simplify the analysis bypassing any potential problems related to surface reconstruction. Despite this advantage, these methods suffer from limited resolution, poor quantification, and are unable to directly measure *M*. They rely on the assumption of *M*-scaling, a principle that could be violated.

Anyway, evidence for the predicted meridional asymmetry is found in a study investigating perceptual filling-in [31] and in a report about the shape of traveling migraine scotoma [32]. Both studies indicate that cortical magnification is not meridional asymmetric but selectively increased on or close to horizontal meridian (HM).

The perceptual filling-in refers to the tendency of stabilized retinal stimuli to fade and become replaced by their background. The time required for this illusion was investigated [31]. It varies with the azimuthal angle *ϕ* of the target in the visual field. The filling-in was facilitated as the target position changed from the horizontal to the vertical meridian. The time was maximal in the horizontal direction and minimal in the vertical. The absolute value differed in the several experiments, which were performed to exclude artifacts. But in general, for a target presented at 8^∘^ eccentricity, the response time for the horizontal condition was more than 30 % longer than that for the vertical one. The author was aware of the fact that his findings can be explained by anisotropic cortical magnification. If magnification on HM is selectively increased, the neural representation of stimuli located on HM is increased too, and the time required for perceptual filling-in is prolonged.

**Fig. 6:**
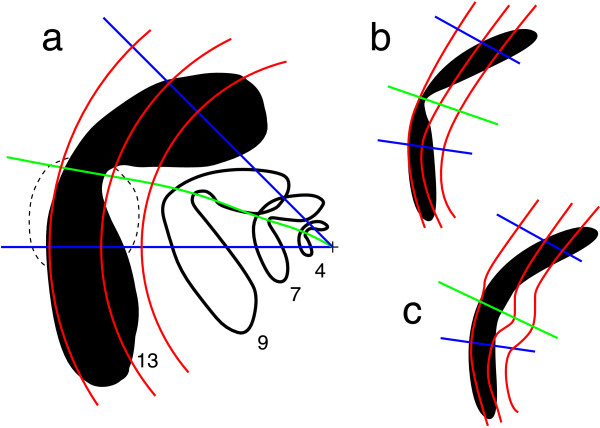
**a** Four subsequent snapshots of a traveling migraine scotoma drawn by Lashley [32]. The *small cross at the left* indicates the center of gaze, and the *numbers* state the time in minutes elapsed before the location of the scotoma was outlined. The point of time when the neurological symptoms were first noticed was chosen as the start. The scotoma travels across the left visual hemifield. **b** and **c** Two different neural patterns in V1 that can explain the scotoma in **a**. Either the noticeable notch in the scotoma pattern along the *green path* in **a** is reflected in the neural pattern by a narrow wave width along the neural representation of the *green path* (**b**) or the retinotopic map is distorted and the wave width is constant (**c**). In this case, the *green path* marks the fundus of the calcarine sulcus

Similar support for selectively increased cortical magnification on HM comes from the shape of traveling scotoma observed during a migraine attack with aura (Fig. 6a). One of the first scientific reports on migraine with aura was given by Lashley [32]. He described in astonishing detail the spatio-temporal patterns of traveling scotoma in his visual field during migraine. The cortical magnification was not considered directly. But the shape of the scotoma in his left visual hemifield clearly hints to meridional asymmetry in *M*. Figure 6a reproduces the spatio-temporal patterns drawn by Lashley. For the purposes of illustration, it was modified: a polar grid was added, the first marked position of the scotoma is not shown, and the latest observed loci of the scotoma is shown as a curve being filled black. This particular shape and location was observed about 13 minutes after the onset of the attack. Two possibly underlying neural patterns are shown in Fig. 6b and c. Assuming a constant speed of 2.5 mm/min of the pathophysiological process within V1, the scotoma at minute 13 (filled) is about 32.5 mm away from the neural representation of the fovea. Mapped with Eq. 3 and the parameter values a=0.117 and b=0.067, this would correspond to 13.6^∘^ degrees eccentricity. This is in sufficient agreement with the position of the retinal blind spot marked by the dashed circular curve.

Independent of the parameter values for *a* and *b* of the monopole map in Eq. 3, the scotoma shown in Fig. 6a cannot be mapped so that its gross shape changes. In other words, the narrow notch along the green path will not disappear. Therefore, either the width of the underlying cortical wave (a spreading depression [45,46]) varies along its path in the cortex (Fig. 6b) or, much more likely, retinotopy is distorted from that of the monopole map (Fig. 6c). The latter is in agreement with our predicted selective increase of cortical magnification along the visual location of the steep fundus of the calcarine sulcus (green path). This provides a test for our 3D model retinotopy. When the selectively increased magnification follows a path not exactly on HM but is shifted to the upper (or lower) field, the neural representation of this path should have the largest length from the neural representation of the fovea to the most anterior part of the retinotopic map. If this path mainly overlaps with the steep fundus, the cortical representation of the horizontal meridian is shifted to the ventral (or dorsal) bank of calcarine sulcus. A qualitative analysis shows that one can measure linear cortical magnification precisely by comparable but simpler drawings of visual migraine aura [47]. Unfortunately, this data was pooled for all meridional directions.

### 4.2 Alternative Tensor Methods

By definition, methods that obtain *M* indirectly suffer from the possibility that the assumption of *M*-scaling locally fails. Only direct methods that precisely delineate the retinotopic organization of human visual can measure cortical magnification. These techniques are mainly used today to model structural and functional variation of the human brain. They can detect group differences, for example, disease effects on cortical anatomy [48,49], but also *M* was investigated [15,17,50]. Problems occur in these studies by smoothing and flattening experimental data, because these techniques are based on mathematical models of retinotopy that introduce certain symmetries (see next section). A recent approach to estimate *M*[18] avoids many of these problems because it is completely data-driven. It preserves geodesic distances by directly computing *M* on the folded cortical surface. They found that *M* is similar in the dorsal and ventral compartments of V1 within each hemisphere. A selective analysis of *M* on HM is not included.

These direct methods to obtain *M* also use a tensor approach to morphometry. They are mainly related to the Laplace–Beltrami operator. One may ask: How do these approaches compare to our tensor description?

In these studies the specific cortical surface parameterization is not essential, though much effort has gone in the development of computationally advantageous parameterizations. If $g_{V1}$ denotes the cortical metric in an arbitrary parameterization, its components can be used to define the Laplace–Beltrami operator as a generalization of the Laplacian differential operator. In contrast, to derive the cortical magnification tensor ***M***, the parameterization is essential. The cortical surface parameterization by sensory coordinates is, in fact, the very idea behind cortical magnification. That is why we have introduced the term cortical expansion tensor ***C*** for this particular form of $g_{V1}$. For other tensor approaches, it is natural and often convenient for the purposes of visualization to parameterize the cortex after inflating it to a sphere using the induced spherical coordinates. One has to define sulcal curves as anchors, e.g., the Talairach coordinate system [51], to achieve that corresponding anatomical landmarks, such as the very cortical sulci, occur at the same spherical coordinate across subjects. The Lagrangian formulation of a receptotopic map is a tensor approach that provides a natural and independent parameterization of the cortical surface. According to this description, data is pooled across subjects in the sensory reference frame, which sort of replaces anatomical anchors by functional labels. A similar idea is used by individual-subject region of interest analysis to identify functional labels [52-54].

### 4.3 Influence of Ocular Dominance

The wedge–dipole model of retinotopy [55] introduces two extensions two the monopole map. It is firstly introduced to model multiple fields (V1–V2–V3 complex) and also to improve the representation of peripheral data. The term “*dipole*” in its name refers to the latter, which is, however, rather irrelevant to our study. The corresponding mathematical changes that also introduce additional parameters are therefore not further considered here. We will only discuss the other extension referred to by the term “*wedge*”. In mathematical terms, wedge refers to a method that compresses the visual hemifield along iso-eccentricity lines (θ=constant) to a wedge with apex angle below *π*. Usually, it is a half disc (apex angle equals π/2). This extension can actually serve two purposes. Firstly, but not relevant in the context of this study, the compression allows a unified model for the topography of the full visual field in areas V1, V2, and V3. Secondly, the compression induces a topographic shear. Such a shear was stated to relate to the combination of two full representations of the visual field into V1 by ocular dominance columns [56,57]. The orientation of the stripes, in which ocular dominance organize and which define the direction of the topographic shear, may well relate to the 3D form of V1.

In primates the patterns of segregated ocular dominance stripes run mostly parallel to the neural representations of *θ* coordinate lines (for the pattern in human, see [58]). At least for non-human primates in a region close to the fovea stripes tend to run horizontally [59]. In the direction perpendicular to the stripes, twice as much cortical space is needed to inject the two ocular representations of the visual field. Therefore, *M* depends on direction. For the sake of simplicity, consider the patterns of ocular dominance stripes running parallel to *θ* coordinate lines in the whole visual field. *M* is usually measured along the *θ* direction perpendicular to the ocular dominance stripes. The value of *M* should be only half as large in opposite direction parallel to the ocular dominance stripes, because only one visual hemifield is mapped along this direction. The effect of such a constant rotational shear, which is not accounted for in our model, is just a scale factor of $\frac{1}{2}$. For example, the flat and curved model of V1 would be half as wide. Since this induces a quasi-conformal map on the flat surface model, the symmetry arguments given above are still valid except for the scaling. However, in the foveal region stripes tend to run horizontally. The opposite scaling effect is obtained when *M* is measured only in this region. If data for *M* is obtained as an average from both regions, the effects can partly be canceled. A detailed analysis of the effect of dominance stripes on cortical magnification and cortical curvature is an open problem.

### 4.4 3D Model of V1 and Its Parameterization

We have made four partly interlinked hypotheses to construct the averaged 3D form of V1, namely that (i) two-thirds of the surface area lie within the CS walls, (ii) the ventral and dorsal half of V1 are symmetric with respect to the fundus, in particular, they extend equally long into the anterior direction, (iii) the shape of the walls can be approximated with smooth profiles (Gaussian-shaped profiles with constant standard deviation), and (iv) the shape of the fundus can also be approximated with a smooth half Gaussian-shaped profile. To obtain a curved retinotopic map on this surface area of V1, three further hypotheses are made: namely, that (v) the horizontal meridian maps to fundus of the calcarine sulcus, (vi) that meridians are equidistantly spaced in the curved V1 (Eq. 12), and that (vii) in all other respects, the retinotopy is governed by the original monopole map.

The hypotheses (i)–(iv) are, to our mind, justified because some smaller deformations within the gross shape of the calcarine sulcus are probably not systematic, and we assume that, on average, their influence is small compared to the curvature effects we investigate in this study. The course of calcarine sulcus varies widely among individuals and the lunate sulcus, which is roughly oriented vertically to it, appears to mark the anterior boundary of V1 in non-human primates, but it is often missing in humans [60]. Autopsy data suggest that V1 proceeds farther anteriorly in the lingual gyrus [8,10], while fMRI data show that the dorsal and ventral compartments of V1 are at least similar in absolute extent [18]. If V1 proceeds farther anteriorly in the lingual gyrus, this can be balanced by higher curvature of this gyrus.

Our main results are robust to some small deviations of these hypotheses, because we only investigate the influence of the gross sulcal pattern on a standard retinotopic layout. For example, we have replaced the Gaussian-shaped profiles ((iii), (iv)) with cosine-shaped profiles with similar results. Furthermore, if (v) holds only approximately and a meridian in the lower or upper quadrant of the visual hemifield with a mild negative or positive shift away from horizontal meridian, respectively, maps onto the fundus, cf. [61], we can still draw our conclusions as to where is the virtual visual streak. In this case, the retinotopy and, in particular, its derivation *M* can still be rather similar in the dorsal and ventral compartments of V1, because hypotheses (v) and (ii) are interlinked and the ventral and dorsal half of V1 (ii) can shift accordingly to respect the major symmetry between upper and lower visual quarter fields [18].

In fact, it provides a test to verify the predicted correlation between anatomical landmarks and functional properties if (v) does not hold exactly, and instead of the horizontal meridian, another path in the visual field, possibly with a variable azimuthal offset with respect to the horizon, maps onto the fundus. Suppose, for example, that a meridional path with a positive shift away from the horizon (upper visual quadrant) is found with an increased cortical magnification, as suggested by data shown in Fig. 6. Then the neural representation of horizontal meridian should be on the ventral bank, because according to our prediction, this path is the location of the fundus in the visual field (if reversed retinotopically mapped). Overall, these hypotheses mainly establish a symmetry between the neural representation of the upper and lower visual field quadrant, as found in [18].

While the 3D form of V1 affects the intrinsic properties of the surface, the layout of the retinal grid on this curved surface ultimately determines ***M***. It has been chosen in a somewhat teleological manner by enforcing that meridians are equidistantly spaced (vi) while at the same time providing more cortex along the fundus that is created by the fold. Although this seems reasonable, one should notice that the location of the virtual visual streak depends on this assumption. Furthermore, because of this assumption, ***M*** is not sensitive to the precise shape of the profiles of the lift function (in particular the Gaussian-shaped profiles with constant standard deviation (iii)). An alternative and completely self-organized method can be provided by neural networks such as a Kohonen net. To model retinotopy on the curved V1, the 2D net of the Kohonen layer must represent the metric of the curved V1 in its lateral connections. The natural learning set is the cell density in the retina. We have investigated such networks and found similar results [37]. The major difference is that the increased areal magnification on the horizontal meridian is shifted slightly into the periphery, where the 3D form of V1 has positive Gaussian curvature. Whereas the maximum areal magnification in this analytic study is found in between the change from negative to positive Gaussian curvature (Fig. 5d).

### 4.5 Development of Cerebral Sulci

On functional grounds, an increased cortical representation of the horizon with respect to the average cortical magnification on meridians (Fig. 5a) serves a similar purpose as a visual streak. It is important to note that our model predicts where a virtual visual streak is located, while the existence of a virtual visual streak is a mathematical consequence of the fact that a surface with non-constant Gaussian curvature cannot be isometrically embedded in one with constant Gaussian curvature (e.g., intrinsically flat surface). Hence, the purpose of our tensor approach is to predict the *shape* and *location* of a virtual visual streak within V1. These are not intrinsic properties of the cortical surface alone but also depend on retinotopy. The virtual visual streak therefore provides a link between cerebral sulci and encephalization of a function because it links anatomy to retinotopy. The virtual visual streak is defined for the visual modality as a selective increase of the number of cortical neurons mapped into the visual domain. It can in principal be defined more generally for all senses and also for a motor function. Such a quantity requires, as cortical magnification does, a notion of distance in the sensory or motor space. For the sensory modalities like visual, auditory, and tactile, the distance between stimuli can be defined based on retina, basilar membrane, and body surface, respectively. The receptor densities must also be considered (det***E*** vs. det***M***). For the sensory modalities like olfactory or also for kinetic modalities, this concept is not readily clear. For example, given two odorants, is it possible to define a notion of distance between them? There is a stereotyped map of odorant receptor inputs in the olfactory bulb [62], but the concept of magnification requires two such maps. Nevertheless, we believe our approach can be applied to all modalities. It may even guide us in the search for a notion of sensory distance for modalities where it is not naturally given.

Animals with a real visual streak have lower encephalization than humans, even with rather lissencephalic cerebral hemispheres [24-30]. When a retina has developed a visual streak, it is not possible to map visual input spatially homogeneous onto a functional field of a lissencephalic cortex without changing the global layout of the map. In other words, the *M*-scaling principle is likely to be locally violated. In such a case (e.g., *Macropus eugenii*), a correlation between retinal ganglion cell density and *M* was found only along VM but not HM [27]. Hence, neural input is increased at the cortical site of the representation of the visual streak. A surface undulation can provide more cortical surface area for this increased input thereby reestablishing the *M*-scaling principle. *Dasyprocta leporina*, another mammalian, in which *M* along HM approximately corresponds to its visual streak, shows gradual encephalization of the visual streak. Nevertheless, its retinotopy has asymmetries that are not directly related to the topography of the retinal ganglion cell density [25].

Irrespective of phylogenetic relationships, species that inhabit open spaces may independently have evolved a visual streak or a virtual visual streak that enables them to better scan the horizon. In other words, if we approach human retinotopy from the comparative standpoint, we can explain why the sulcal position is where it is, though not necessarily how sulci develop. The specific location of CS might be a convergent evolutionary solution to enhance visual function on HM. The human retina poses a very mild visual streak [19,21] and even without taking the visual streak into account, the *M*-scaling is violated in all current theoretical models of human retinotopic maps, because they have a decreased cortical representation of the horizon with respect to the average cortical magnification on meridians. At the location where *M*-scaling is violated either less or more cortical surface area is available to process information from a certain number of retinal ganglion cells. Therefore, our predicted selective increase of cortical magnification due to cortical folding, i.e., the virtual visual streak with selectively increased det***E***, indicates a form of encephalized visual magnification. If the virtual visual streak is actually a phylogenetic shadow of a visual streak in human ancestors, which is supported by its mild occurrence [19,21], thus it is not an independent evolutionary solution: a switch from selectively increased retinal to cortical magnification on HM took place.

To summarize, the theme from structure to function is central to biology, as is the reverse direction from function to structure. We doubt that the precise correlation between the primary visual cortex, as an functional area, and the calcarine sulcus, as an anatomical structure, occurred by chance.

None of the existing mathematical models is suited to describe retinotopy in relation to the cortical geometry. Most of them are in fact either 1D functions assuming an inverse linear mapping given by two parameters (*a*, *b* or *A*, $E_{2}$, see Eq. 3), without taking into account that a 2D generalization is not trivial, or they are intrinsically flat maps described by an analytical function. The monopole map and its five-parameter extension, called wedge-dipole map [55], are by definition conformal and quasi-conformal, respectively. Any analytic function has a high inner structure, i.e., exhibits certain symmetries. Alternative nonconformal maps suffer from inconsistencies with the definition *M*[63]. It is assumed that single components of the Jacobian matrix $J_{\Phi }$ (Eq. 5) scale with *M*. But $J_{\Phi }$, unlike ***M***, contains a rotation carrying the retinal directions onto its neural representations. This renders the assumption implausible if conformal mapping is not presumed in the first place.

Generally, when cortical functions became encephalized and even completely new sensory and motor areas simultaneously arose, this constituted a developmental force for the prominent patterns in the highly convoluted surface of the cerebral cortex in humans. Linking cortical magnification to anatomy with methods of continuum mechanics and complex analysis may shed some light on the underlying pattern formation principles.

## Competing interests

The authors declare that they have no competing interests.

## Authors’ contributions

MAD conceived and analysed the mathematical model, and wrote the paper. JT and MAD developed and implemented the simulation, and read and approved the final manuscript.

## References

[B1] Rajkowska G Goldman-Rakic PS Cytoarchitectonic definition of prefrontal areas in the normal human cortex: II. Variability in locations of areas 9 and 46 and relationship to the Talairach coordinate system Cereb Cortex 1995 5 4 323 337 10.1093/cercor/5.4.323 7580125

[B2] Thompson PM Schwartz C Lin RT Khan AA Toga AW Three-dimensional statistical analysis of sulcal variability in the human brain J Neurosci 1996 16 13 4261 4274 8753887 10.1523/JNEUROSCI.16-13-04261.1996 PMC6578992

[B3] Roland P Zilles K Structural divisions and functional fields in the human cerebral cortex Brains Res Rev 1998 26 87 105 10.1016/S0165-0173(97)00058-1 9651489

[B4] Amunts K Schleicher A Burgel U Mohlberg H Uylings HB Zilles K Broca’s region revisited: cytoarchitecture and intersubject variability J Comp Neurol 1999 412 2 319 341 10.1002/(SICI)1096-9861(19990920)412:2<319::AID-CNE10>3.0.CO;2-7 10441759

[B5] Lotze M Erb M Flor H Huelsmann E Godde B Grodd W fMRI evaluation of somatotopic representation in human primary motor cortex NeuroImage 2000 11 5 473 481 10.1006/nimg.2000.0556 10806033

[B6] Gaschler-Markefski B Baumgart F Tempelmann C Schindler F Stiller D Heinze HJ Scheich H Statistical methods in functional magnetic resonance imaging with respect to nonstationary time-series: auditory cortex activity Magn Reson Med 1997 38 5 811 820 10.1002/mrm.1910380518 9358456

[B7] Rademacher J Morosan P Schormann T Schleicher A Werner C Freund HJ Zilles K Probabilistic mapping and volume measurement of human primary auditory cortex NeuroImage 2001 13 4 669 683 10.1006/nimg.2000.0714 11305896

[B8] Stensaas SS Eddington DK Dobelle WH The topography and variability of the primary visual cortex in man J Neurosurg 1974 40 6 747 755 10.3171/jns.1974.40.6.0747 4826600

[B9] Gilissen E Zilles K The calcarine sulcus as an estimate of the total volume of human striate cortex: a morphometric study of reliability and intersubject variability J Hirnforsch 1996 37 57 66 8964978

[B10] Andrews TJ Halpern SD Purves D Correlated size variations in human visual cortex, lateral geniculate nucleus, and optic tract J Neurosci 1997 17 2859 2868 9092607 10.1523/JNEUROSCI.17-08-02859.1997 PMC6573115

[B11] Watson JD Myers R Frackowiak RS Hajnal JV Woods RP Mazziotta JC Shipp S Zeki S Area V5 of the human brain: evidence from a combined study using positron emission tomography and magnetic resonance imaging Cereb Cortex 1993 3 2 79 94 10.1093/cercor/3.2.79 8490322

[B12] Walters NB Egan GF Kril JJ Kean M Waley P Jenkinson M Watson JDG In vivo identification of human cortical areas using high-resolution MRI: an approach to cerebral structure-function correlation Proc Natl Acad Sci USA 2003 100 5 2981 2986 10.1073/pnas.0437896100 12601170 PMC151452

[B13] Schneider W Noll DC Cohen JD Functional topographic mapping of the cortical ribbon in human vision with conventional MRI scanners Nature 1993 365 6442 150 153 10.1038/365150a0 8371756

[B14] Engel SA Rumelhart DE Wandell BA Lee AT Glover GH Chichilnisky EJ Shadlen MN fMRI of human visual cortex Nature 1994 369 525 10.1038/369525a0 8031403

[B15] Sereno MI Dale AM Reppas JB Kwong KK Belliveau JW Brady TJ Rosen BR Tootell RB Borders of multiple visual areas in humans revealed by functional magnetic resonance imaging Science 1995 268 889 893 10.1126/science.7754376 7754376

[B16] DeYoe EA Carman GJ Bandettini P Glickman S Wieser J Cox R Miller D Neitz J Mapping striate and extrastriate visual areas in human cerebral cortex Proc Natl Acad Sci USA 1996 93 6 2382 2386 10.1073/pnas.93.6.2382 8637882 PMC39805

[B17] Engel SA Glover GH Wandell BA Retinotopic organization in human visual cortex and the spatial precision of functional MRI Cereb Cortex 1997 7 2 181 192 10.1093/cercor/7.2.181 9087826

[B18] Qiu A Rosenau BJ Greenberg AS Hurdal MK Barta P Yantis S Miller MI Estimating linear cortical magnification in human primary visual cortex via dynamic programming NeuroImage 2006 31 125 138 10.1016/j.neuroimage.2005.11.049 16469509

[B19] Stone J Johnston E The topography of primate retina: a study of the human, bushbaby, and new- and old-world monkeys J Comp Neurol 1981 196 2 205 223 10.1002/cne.901960204 7217355

[B20] Wässle H Grünert U Röhrenbeck J Boycott BB Cortical magnification factor and the ganglion cell density of the primate retina Nature 1989 341 6243 643 646 10.1038/341643a0 2797190

[B21] Curcio CA Allen KA Topography of ganglion cells in human retina J Comp Neurol 1990 300 5 25 10.1002/cne.903000103 2229487

[B22] Rovamo J Virsu V Isotropy of cortical magnification and topography of striate cortex Vis Res 1984 24 3 283 286 10.1016/0042-6989(84)90133-0 6719844

[B23] Rajimehr R Tootell RB Does retinotopy influence cortical folding in primate visual cortex? J Neurosci 2009 29 36 11149 11152 10.1523/JNEUROSCI.1835-09.2009 19741121 PMC2785715

[B24] Steinberg RH Reid M Lacy PL The distribution of rods and cones in the retina of the cat (*Felis domesticus*) J Comp Neurol 1973 148 2 229 248 10.1002/cne.901480209 4700509

[B25] Picanco-Diniz CW Silveira LC de Carvalho MS Oswaldo-Cruz E Contralateral visual field representation in area 17 of the cerebral cortex of the agouti: a comparison between the cortical magnification factor and retinal ganglion cell distribution Neuroscience 1991 44 2 325 333 10.1016/0306-4522(91)90057-U 1944888

[B26] Peichl L Topography of ganglion cells in the dog and wolf retina J Comp Neurol 1992 324 4 603 620 10.1002/cne.903240412 1385496

[B27] Vidyasagar TR Wye-Dvorak J Henry GH Mark RF Cytoarchitecture and visual field representation in area 17 of the tammar wallaby (*Macropus eugenii*) J Comp Neurol 1992 325 2 291 300 10.1002/cne.903250211 1281175

[B28] Guo X Sugita S Topography of ganglion cells in the retina of the horse J Vet Med Sci 2000 62 11 1145 1150 10.1292/jvms.62.1145 11129856

[B29] Arrese CA Rodger J Beazley LD Shand J Topographies of retinal cone photoreceptors in two Australian marsupials Vis Neurosci 2003 20 3 307 311 14570252 10.1017/s0952523803203096

[B30] Calderone JB Reese BE Jacobs GH Topography of photoreceptors and retinal ganglion cells in the spotted hyena (*Crocuta crocuta*) Brain Behav Evol 2003 62 4 182 192 10.1159/000073270 14573992

[B31] Sakaguchi Y Visual field anisotropy revealed by perceptual filling-in Vis Res 2003 43 19 2029 2038 10.1016/S0042-6989(03)00305-5 12842156

[B32] Lashley K Patterns of cerebral integration inicated by scotomas of migraine Arch Neurol Psychiatry 1941 46 331 339 10.1001/archneurpsyc.1941.02280200137007

[B33] Janik J Ropella K DeYoe E Distortions of human retinotopy obtained with temporal phase mapped fMRI Soc Neurosci Abstr 2003 29 Article ID 658.8

[B34] Chung MK Worsley KJ Robbins S Paus T Taylor J Giedd JN Rapoport JL Evans AC Deformation-based surface morphometry applied to gray matter deformation NeuroImage 2003 18 2 198 213 10.1016/S1053-8119(02)00017-4 12595176

[B35] Henriksson L Karvonen J Salminen-Vaparanta N Railo H Vanni S Retinotopic maps, spatial tuning, and locations of human visual areas in surface coordinates characterized with multifocal and blocked fMRI designs PLoS ONE 2012 7 5 Article ID e36859 10.1371/journal.pone.0036859 PMC3348898 22590626

[B36] Salencon J Handbook of Continuum Mechanics 2001 Springer, Berlin

[B37] Tusch J: **Simulation of partial visual field defects using selforganizing maps of the curved surface of the primary visual cortex**. *Master’s thesis*. Otto-von-Guericke University of Magdeburg, Magdeburg; 2004.

[B38] Fischer B Overlap of receptive field centers and representation of the visual field in the cat’s optic tract Vis Res 1973 13 11 2113 2120 10.1016/0042-6989(73)90188-0 4763524

[B39] Daniel PM Whitteridge D The representation of the visual field in striate and adjoining cortex of the owl monkey (*Aotus trivirgatus*) J Physiol 1961 159 203 22113883391

[B40] Schwartz E Spatial mapping in primate sensory projection: analytic structure and relevance to perception Biol Cybern 1977 25 181 194 10.1007/BF01885636 843541

[B41] Ahlfors LV Complex Analysis 1953 McGraw-Hill, New York

[B42] Slotnick SD Klein SA Carney T Sutter EE Electrophysiological estimate of human cortical magnification Clin Neurophysiol 2001 112 7 1349 1356 clinical trial 10.1016/S1388-2457(01)00561-2 11516748

[B43] Cowey A Rolls ET Human magnification factor and its relation to visual acuity Exp Brain Res 1974 3 447 454 4442497 10.1007/BF00237163

[B44] Kao C-Y Hofer M Sapiro G Stern J Rehm K Rottenberg DA A geometric method for automatic extraction of sulcal fundi IEEE Trans Med Imag 2007 26 4 530 540 doi:10.1109/TMI.2006.886810 10.1109/TMI.2006.886810 17427740

[B45] Dahlem MA Müller SC Migraine aura dynamics after reverse retinotopic mapping of weak excitation waves in the primary visual cortex Biol Cybern 2003 88 419 424 12789490 10.1007/s00422-003-0405-y

[B46] Dahlem MA Hadjikhani N Migraine aura: retracting particle-like waves in weakly susceptible cortex PLoS ONE 2009 4 Article ID e5007 10.1371/journal.pone.0005007 PMC2659426 19337363

[B47] Grüsser OJ Migraine phosphenes and the retino-cortical magnification factor Vis Res 1995 35 1125 1134 10.1016/0042-6989(94)00187-Q 7762167

[B48] Baseler HA Brewer AA Sharpe LT Morland AB Jagle H Wandell BA Reorganization of human cortical maps caused by inherited photoreceptor abnormalities Nat Neurosci 2002 5 4 364 370 10.1038/nn817 11914722

[B49] Van Essen DC Dierker D Snyder AZ Raichle ME Reiss AL Korenberg J Symmetry of cortical folding abnormalities in Williams syndrome revealed by surface-based analyses J Neurosci 2006 26 5470 5483 10.1523/JNEUROSCI.4154-05.2006 16707799 PMC6675292

[B50] Duncan R Boynton G Cortical magnification within human primary visual cortex correlates with acuity thresholds Neuron 2003 38 659 671 10.1016/S0896-6273(03)00265-4 12765616

[B51] Talairach J Tournoux P Co-planar Stereotaxic Atlas of the Human Brain 1988 Thieme, New York

[B52] Kanwisher N McDermott J Chun MM The fusiform face area: a module in human extrastriate cortex specialized for face perception J Neurosci 1997 17 11 4302 4311 9151747 10.1523/JNEUROSCI.17-11-04302.1997 PMC6573547

[B53] Epstein R Kanwisher N A cortical representation of the local visual environment Nature 1998 392 6676 598 601 10.1038/33402 9560155

[B54] Downing P Chan A Peelen M Dodds C Kanwisher N Domain specificity in visual cortex Cereb Cortex 2005 16 10 1453 1461 10.1093/cercor/bhj086 16339084

[B55] Balasubramanian M Polimeni J Schwartz E The V1–V2–V3 complex: quasiconformal dipole maps in primate striate and extra-striate cortex Neural Netw 2002 15 1157 1163 10.1016/S0893-6080(02)00094-1 12425434

[B56] Tootell RB Silverman MS Switkes E De Valois RL Deoxyglucose analysis of retinotopic organization in primate striate cortex Science 1982 218 4575 902 904 10.1126/science.7134981 7134981

[B57] Sakitt B Why the cortical magnification factor in rhesus can not be isotropic Vis Res 1982 22 3 417 421 10.1016/0042-6989(82)90158-4 7090196

[B58] Horton JC Hedley-Whyte ET Mapping of cytochrome oxidase patches and ocular dominance columns in human visual cortex Philos Trans R Soc Lond B, Biol Sci 1984 304 255 272 10.1098/rstb.1984.0022 6142485

[B59] LeVay S Connolly M Houde J Van Essen DC The complete pattern of ocular dominance stripes in the striate cortex and visual field of the macaque monkey J Neurosci 1985 5 2 486 501 3973679 10.1523/JNEUROSCI.05-02-00486.1985 PMC6565187

[B60] Connolly CJ External Morphology of the Primate Brain 1950 Thomas, Springfield

[B61] Aine CJ Supek S George JS Ranken D Lewine J Sanders J Best E Tiee W Flynn ER Wood CC Retinotopic organization of human visual cortex: departures from the classical model Cereb Cortex 1996 6 354 361 10.1093/cercor/6.3.354 8670663

[B62] Buck LB Olfactory receptors and odor coding in mammals Nutr Rev 2004 62 S184 S188 15630933 10.1111/j.1753-4887.2004.tb00097.x

[B63] Dayan P Abbott LF Theoretical Neuroscience: Computational and Mathematical Modeling of Neural Systems 2001 MIT Press, Cambridge

